# The Effect of Acute *Rhodiola rosea* Ingestion on Exercise Heart Rate, Substrate Utilisation, Mood State, and Perceptions of Exertion, Arousal, and Pleasure/Displeasure in Active Men

**DOI:** 10.1155/2014/563043

**Published:** 2014-04-27

**Authors:** Michael J. Duncan, Neil D. Clarke

**Affiliations:** ^1^Department of Biomolecular and Sports Sciences, Coventry University, Coventry CV1 5HB, UK; ^2^Human Performance Laboratory, Department of Biomolecular and Sports Sciences, Coventry University, James Starley Building, Priory Street, Coventry CV 5HB, UK

## Abstract

The aim of this study was to examine the effect of acute *Rhodiola rosea* (*R. rosea*) ingestion on substrate utilisation, mood state, RPE, and exercise affect. Ten males (mean age ± S.D. = 26 ± 6 years) completed two 30-minute cycling trials at an intensity of 70% of $\dot{\text{V}}{\text{O}}_{2\max}$ following ingestion of either 3 mg·kg^−1^ body mass of *R. rosea* or placebo using a double-blind, crossover design. During exercise, heart rate and RPE were recorded. Participants completed measures of mood state and exercise affect before and after exercise. Expired air samples were taken during exercise to determine substrate utilisation. Repeated measures analysis of variance indicated that RPE was significantly lower at 30 minutes into exercise versus placebo (*P* = 0.003). Perceptions of arousal (*P* = 0.05) and pleasure were significantly higher after exercise with *R. rosea* compared to placebo (*P* = 0.003). Mood state scores for vigor were also higher in *R. rosea* condition compared to placebo (*P* = 0.008). There were no significant differences in energy expenditure, carbohydrate, or fat oxidation between conditions (*P* > 0.05). Ingestion of *R. rosea* favourably influenced RPE and exercise affect without changes in energy expenditure or substrate utilization during 30-minute submaximal cycling performance.

## 1. Introduction

*Rhodiola rosea* (*R. rosea*) is an herb that grows in mountainous regions of North America, Europe, and Asia. It has been used in traditional folk medicine for centuries as a treatment for fatigue and mood disorders [1].* R. rosea* has been extensively studied by scientists in the former Soviet Union and has predominantly been found to result in favourable effects on exercise performance [2]. However, this prior work has been lacking in experimental control [3]. Some recent studies have supported the results of earlier work by identifying antioxidant and anti-inflammatory properties of* R. rosea* [3, 4]. Another work has suggested ingestion of* R. rosea* appears to be effective, either acutely [5] or with daily supplementation [6], for reducing perceived fatigue and improving cognition [5, 7], as well as reducing markers of physiological and psychological stress [7, 8].

Although* R. rosea* ingestion has been identified as a means to reduce physical fatigue, its efficacy during exercise is unclear. Animal based research has shown increased swim time to exhaustion in rats [3, 9] but the impact of* R. rosea* ingestion on exercise performance in humans is equivocal. Some studies have shown no effect of* R. rosea* ingestion on exercise performance [4, 10–12] whilst others have supported its use [13, 14]. The array of protocols and supplementation doses used in prior studies has also limited comparisons between studies and provides one potential explanation for the discrepancy in their findings. For example, de Bock et al. [13] reported that an acute dose of* R. rosea* increased time to exhaustion during an incremental cycle ergometer protocol. Conversely, in the same study, 4 weeks of* R. rosea* supplementation did not significantly improve cycling performance. This was the first study to show an acute impact of* R. rosea* ingestion on human exercise performance. More recent research by Noreen et al. [14] examined the efficacy of a 3 mg·kg^−1^ body mass dose of* R. rosea* on 6-mile cycle time trial performance in 18 active women. They reported that* R. rosea* significantly decreased submaximal exercise heart rate, reduced RPE, and improved time trial performance time. Noreen et al. [14] subsequently suggested that acute* R. rosea* ingestion might be employed by active individuals while exercising to complete a set distance in the shortest time.

Such data are interesting; as if* R. rosea* does positively impact on exercise, it may be safe and legal way to enhance exercise performance in athletes and the exercise experience in recreational gym users. The mechanism by which* R. rosea* might improve performance is also unclear. Abidov et al. [15] demonstrated that an extract of* R. rosea* significantly prolonged the duration of exhaustive swimming in rats and stimulated ATP synthesis in muscle during exercise. These findings led Perfumi and Mattioli [16] to speculate that, in addition to enhancing the catecholaminergic system, the ability of* R. rosea* to increase performance involves an improvement in cellular energy metabolism. However, some studies have suggested that* R. rosea* acts to acutely increase endogenous opioid production or receptor sensitivity [2, 13] which subsequently impacts on brain dopamine and serotonin and cardiac activity and attenuates the perception of effort at a given workload [14]. However, as only two studies have examined acute* R. rosea* ingestion on exercise performance to date, further data is needed on this topic. Moreover, both de Bock et al. [13] and Noreen et al. [14] employed performance based protocols to examine the effect of* R. rosea* ingestion. While these are valuable in understanding the effect of a nutritional supplement on performance responses, different protocols may be better placed to evidence any effect of* R. rosea* on exercise performance. For example, using steady state submaximal exercise in the presence of* R. rosea* and placebo would allow for power output to remain constant across a given period. This then allows for better like-for-like comparison of data between the active supplement and placebo trials. Moreover, when employing protocols where intensity is modified throughout (as is the case with both time trials and time to exhaustion) understanding any impact of the exercise protocol of substance ingested becomes more difficult because even small increases in exercise intensity have been shown to markedly change feelings of pleasure during exercise [17].

Furthermore, given the postulation that* R. rosea* acts through increased opioid production, it is perhaps surprising that few studies have examined its impact on affect. Noreen et al. [14] reported no effect of* R. rosea* ingestion on mood state, as assessed using the POMS questionnaire. However, changes in mood state with exercise have been reported following ingestion of a range of substances including caffeine [18, 19] and carbohydrate [20]. Hedonic theory [21] postulates that hedonic responses (i.e., pleasure versus displeasure) following a behaviour influence decisions regarding whether or not to repeat that behaviour. As a consequence if nutritional manipulation enhances the pleasure response to exercise, according to hedonic theory, it may mean individuals are more likely to exercise [21]. This is important as different exercise intensities and individual fitness will create variability in the affective response to exercise [22–24] and the effect of nutritional manipulation on these responses is not well known. Changes in mood and feelings of pleasure may be one reason why individuals do not participate in regular exercise and physical activity [19] and favourable affective states benefit exercise performance [25]. Research by DaSilva et al. [26] has also identified that affective and perceptual responses to exercise differ depending on adiposity status. Assuming that the affective response to exercise is the same in recreational exercises compared to high level performers may therefore lead to erroneous conclusions regarding the exercise-affect relationship. Thus, while the extant literature examining the effect of* R. rosea* ingestion has focused on performance measures, the current study seeks to develop this work by employing a model that can be applied to recreational exercisers and is more applicable to the exercising public in general.

The aim of this study was to examine the effect of acute* R. rosea* ingestion on substrate utilisation, mood state, RPE, perceptions of arousal, and pleasure/displeasure in a population of recreationally active men. It was hypothesized that ingestion of* R. rosea* would positively influence perceived exertion during exercise as well as enhancing mood and perceptions of arousal and pleasure/displeasure before and after cycling exercise. Testing these hypotheses will address gaps in knowledge on the effect of* R. rosea* by examining their effects on an active but not performance oriented group, more typical of regularly active individuals in the general population. The study will also combine physiological and psychological measures to address gaps in the literature base by assessing substrate utilisation, heart rate, RPE, mood state, and perceptions of arousal and pleasure/displeasure to provide a multidisciplinary investigation of the effect of* R. rosea* during exercise.

## 2. Method

### 2.1. Design

This study employed a within-participants double-blind cross-over design whereby participants visited the laboratory on 3 occasions at the same time of day in a well-rested and well-hydrated state. Ten male, recreational exercisers (mean age ± S.D. = 26 ± 6 years) participated in this study. All participants were asked to refrain from vigorous exercise and maintain normal dietary patterns in the 48 hours prior to testing and were asked not to consume caffeine for 24 hours before testing. During the first visit participants completed a familiarization session with the measures to be used in the subsequent experimental trials and completed an incremental exercise test to assess $\dot{\text{V}}{\text{O}}_{2\max}$. Inclusion criteria included being male and habitually engaged in recreational physical activity of more than 3 but less than 10 hours per week and not including formal competitive sports performance. Participants were excluded if they had a musculoskeletal or cardiovascular contraindication to exercise, were taking any medication that could impact mood/affect, engaged in less than 3 or more than 10 hours physical activity per week, or were engaged in competitive sports activity as part of their habitual physical activity.

The incremental exercise test was performed on a mechanically braked cycle ergometer (Monark Exercise AB, Sweden) to assess $\dot{\text{V}}{\text{O}}_{2\max}$ following previously published guidelines [27]. Initially, participants were fitted to the ergometer and performed a 5 min warm-up at 25 Watts. The initial work rate for the test was 80 Watts with the work rate increasing by 40 Watts every 3 minutes. This protocol, using 3-minute stages rather than a ramp protocol, was employed based on prior research suggesting that this form of protocol provides more reliable physiological measures, particular for the population characteristics examined in this study (i.e., active but not cycling trained specifically) [27]. Participants were asked to maintain their pedal cadence at 70 rpm and were given visual feedback from the Monark control box in order to do this. Expired air was collected via the Douglas bag technique during the final minute of each incremental exercise stage. Samples were analyzed for oxygen and carbon dioxide content (Servomex, Crowborough, England) and expired air volume (Harvard dry gas meter, Harvard Apparatus, Kent, England) with values for oxygen consumption ($\dot{\text{V}}{\text{O}}_{2}$) and carbon dioxide production ($\dot{\text{V}}{\text{CO}}_{2}$) subsequently calculated. Heart rate (Polar Electro, Kempele, Finland) and rating of perceived exertion (RPE), using the Borg 6–20 RPE scale [28], were recorded during the final 15 seconds of each workload. Exercise was continued to exhaustion in order for measurement of $\dot{\text{V}}{\text{O}}_{2\max}$ to be made. Participants were judged to have reached $\dot{\text{V}}{\text{O}}_{2\max}$ if they presented at least 3 of the following: (a) a respiratory exchange ratio of greater than 1.1, (b) a heart rate during the last stage of testing that was ±10 beats of age predicted maximum heart rate, (c) an RPE of 18 or greater, (d) a plateau in VO_2_ with an increase in workload, and (e) volitional fatigue. These criteria were employed based on those commonly used for maximal oxygen uptake [29]. All participants met these criteria during their incremental exercise test. Mean ± S.D. of participants' baseline $\dot{\text{V}}{\text{O}}_{2\max}$ values was 50.5 ± 6.6 mL·kg^−1^·min^−1^ (range: 38.8–58.4 mL·kg^−1^·min^−1^). Descriptive information for the participants is presented in Table 1.

### 2.2. Experimental Protocol

On completion of the $\dot{\text{V}}{\text{O}}_{2\max}$ testing and following a period of at least 72 hours participants completed two 30-minute submaximal cycling trials at a workload of 70% $\dot{\text{V}}{\text{O}}_{2\max}$ in a fasted state. Self-report of dietary intake was employed to assess and control dietary intake in the 24 hours prior to exercise trials. This was used to ensure that the same/similar per testing meals were consumed prior to experimental trials and that caffeine and alcohol had not been consumed in the 24 hours prior to testing. The duration and intensity of exercise were chosen as it sits within the American College of Sports Medicine guidelines for the prescription of exercise for health benefit [30]. On a molecular basis, the main active ingredients of* R. rosea* appear to be Tyrosol and its glucoside known as salidroside and following oral ingestion of 100 mg·kg^−1^ salidroside, the half-life of salidroside appears to be 1.32 ± 0.22 h [31]. Therefore, conditions were randomised, separated by 48–72 hours, and consisted of a* R. rosea* condition where 3 mg·kg^−1^ body mass of* R. rosea* (Indigo Herbs, Glastonbury, UK) placed in a coloured, opaque gelatin capsule or a placebo (3 mg·kg^−1^ body mass of maltodextrin, (MyProtein, Northwich, UK)) was ingested with 250 mL water. The capsules were indistinguishable between* R. rosea* and placebo conditions and were ingested 60 min before exercise on an empty stomach. The amount of total maltodextrin ingested was approximately 170 mg and thus highly unlikely to have had any impact on exercise performance or metabolism [14]. The dose of* R. rosea* used was based on the previous work of de Bock et al. [13] and Noreen et al. [14] who observed improvements in endurance performance following acute ingestion of* R. rosea*.

During each of the cycling trials, heart rate was monitored (Polar RS400, Polar Electro Oy, Kempele, Finland) continuously and assessed every 10 minutes. Participants were also asked to provide ratings of perceived exertion (RPE) using the Borg 6–20 scale [28]. Prior to commencing the experimental trials the memory-anchoring approach [32] was employed whereby participants were asked to remember a time when they were at rest (attributed to RPE of 6) and a time when they had reached a level of exertion that was maximal attributed to RPE of 20. The RPE scale was presented to students for 1 minute prior to their rating of perceived exertion at each time point and was not visible other than this. Prior to any substance ingestion, 60 min after ingestion (at the onset of each exercise bout) and immediately on completion of each exercise bout, participants also completed the feeling scale (FS) as a measure of pleasure and displeasure [33]. This is an 11-item single-item scale ranging from +5 (very good) to −5 (very bad) that is used to quantify pleasure/displeasure. The Felt Arousal Scale (FAS) was also used as a measure of arousal [34]. This is a six-item scale ranging from 1 (low arousal) to 6 (high arousal). Mood state was also assessed using the fatigue and vigour subscales of the Brunel Mood State Inventory (BRUMS) [35]. This is a well-established, reliable, and valid measure of mood state that has been previously employed to assess the mood state response to various exercise modes [18, 35, 36]. The fatigue and vigor subscales were chosen in particular as prior research [1, 7] has suggested* R. rosea* may particularly influence feelings of fatigue and/or vigor.

Participants were introduced to these scales during their first visit to the laboratory (prior to establishment of $\dot{\text{V}}{\text{O}}_{2\max}$). Standardised instructions for completing the FS and FAS were read to participants at the beginning of each trial.

## 3. Substrate Oxidation

Prior to cycling (but whilst seated on the exercise bike) at 14 min and 29 min during each exercise bout expired air samples were collected using the Douglas bag technique to estimate fat and carbohydrate (CHO) oxidation rates. The collection time was 60 s and samples were analysed for oxygen and carbon dioxide content (Servomex, Crowborough, England) that was calibrated at two points before each assessment using gases of known concentrations (15.12% O_2_, 5.1% CO_2_) in accordance with manufacturers guidelines and expired air volume (Harvard dry gas meter, Harvard Apparatus, Kent, England) with values for oxygen consumption ($\dot{\text{V}}{\text{O}}_{2}$) and carbon dioxide production ($\dot{\text{V}}{\text{CO}}_{2}$) subsequently calculated. Energy expenditure (Kcal*·*min^−1^) and the oxidation rates for carbohydrate and fat (g·min^−1^) were then calculated according to the Weir [37] and Frayn [38] equations, respectively.

### 3.1. Statistical Analysis

Data were analysed in a number of ways. A series of 2 (substance ingested) × 4 (time point) ways repeated measures analysis of variance was used to examine any differences in heart rate and RPE. A series of 2 (substance ingested) X 3 (time point, before ingestion, after ingestion but before exercise, and after exercise) ways repeated measures analysis of variance was used to examine any differences in mood state and perceptions of arousal and pleasure/displeasure and substrate utilisation. Where any significant differences were discovered, Bonferroni pairwise multiple comparisons were used to determine where the differences lay. Partial *η* ^2^ was used as a measure of effect size, statistical significance was set at *P* = 0.05 a priori, and the Statistical Package for Social Sciences (Version 20) was used for all analysis (SPSS Inc., IL, USA).

## 4. Results

In respect to heart rate, results indicated no significant interactions or main effect due to the substances ingested. There was however a significant main effect for time (*F* 3, 27 = 266.7, *P* = 0.0001, *Pη* ^2^ = 0.967) where heart rate was significantly increased throughout the exercise bout irrespective of substance ingested. Mean ± SE of heart rate (bpm) was 77 ± 4 at rest compared to 151 ± 4, 158 ± 4, and 162 ± 4 at 10, 20, and 30 minutes into the exercise bout, respectively. Bonferroni comparisons indicated that heart rate was significantly different at all time points (*P* = 0.001 in all cases).

**Figure 1:**
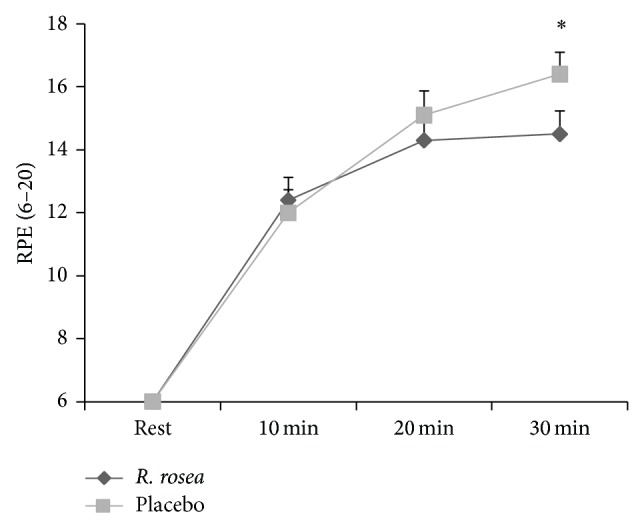
Mean ± SE of RPE (6–20) at rest and during 30 min submaximal cycling between* R. rosea* and placebo conditions (^*^ *P* = 0.003).

In the case of RPE, results indicated a significant substance × time interaction (*F* 3, 27 = 6.12, *P* = 0.003, *Pη* ^2^ = 0.405, see Figure 1). RPE data were not significantly different at rest and at 10 and 20 minutes into the exercise bout between the two conditions. However, RPE in the placebo condition was significantly higher than that in the* R. rosea* condition 30 minutes into the exercise bout (*P* = 0.003).

Scores from the fatigue subscale of the BRUMS indicated a significant main effect across time (*F* 2, 18 = 12.879, *P* = 0.0001, *Pη* ^2^ = 0.589) where scores were significantly higher after exercise compared to before exercise (*P* = 0.028) and compared to before exercise but after ingestion (*P* = 0.008). There were no other significant main effects of interactions for fatigue subscale data. In regard to the vigor subscale of the BRUMS, there was no significant substance × time interaction. There were however significant main effects for time (*F* 2, 18 = 4.435, *P* = 0.027, *Pη* ^2^ = 0.350) and for the substance ingested (*F* 2, 9 = 11.692, *P* = 0.008, *Pη* ^2^ = 0.565). Scores for vigor were significantly lower at after exercise compared to after ingestion but before exercise (*P* = 0.04). Mean ± SE for vigor scores was 6.9 ± 0.56 before ingestion, 7.4 ± 0.44 after ingestion but before exercise, and 5.6 ± 0.74 after exercise. For the substance ingested, scores for vigor were significantly higher in the* R. rosea* condition (*P* = 0.008) compared to the placebo. Mean ± SE for vigor was 7.6 ± 0.52 in the* R. rosea* condition compared to 5.7 ± 0.57 in the placebo condition.

**Figure 2:**
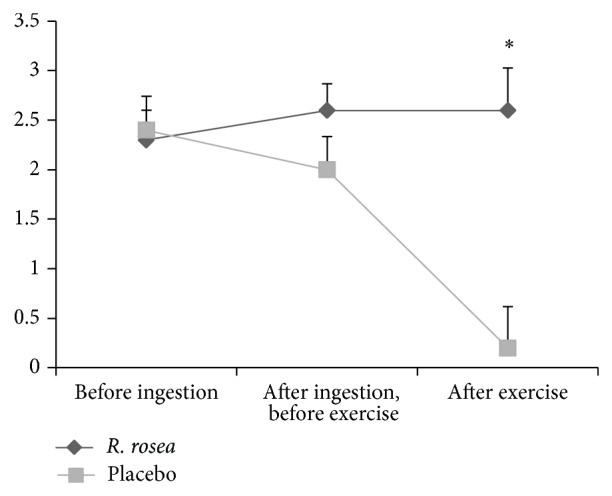
Mean ± SE of perception of pleasure/displeasure before ingestion, after ingestion but before exercise, and after exercise between* R. rosea* and placebo conditions (^*^ *P* = 0.003).

Perceptions of arousal were also significantly different in the presence of* R. rosea* compared to placebo (*F* 1, 9 = 5.0, *P* = 0.05, *Pη* ^2^ = 0.357) with scores for arousal being higher (3.1 ± 0.19) in the presence of* R. rosea *compared to placebo (2.8 ± 0.11). When data for feeling of pleasure/displeasure were assessed, there was however a significant condition × time effect (*F* 2, 18 = 12.795, *P* = 0.0001, *Pη* ^2^ = 0.587, see Figure 2). This followed a similar pattern to RPE with no significant difference evident before ingestion (*P* = 0.591) or after ingestion but before exercise (*P* = 0.08). There was however significantly lower scores for pleasure after exercise in the placebo condition compared to the* R. rosea* condition (*P* = 0.003).

**Figure 3:**
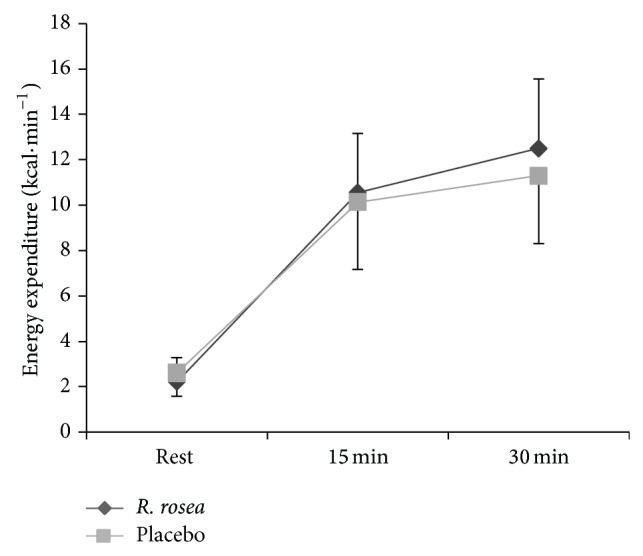
Mean ± S.D. energy expenditure (Kcal·min^−1^) during 30 min submaximal cycling between* R. rosea* and placebo conditions.

**Figure 4:**
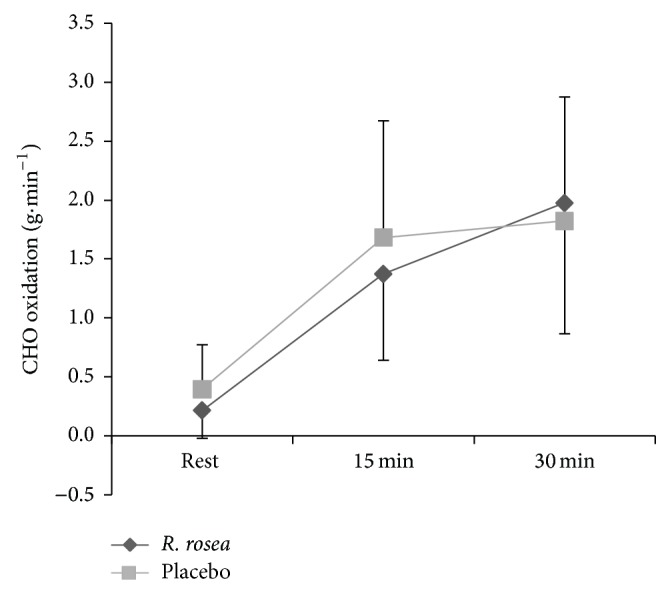
Mean ± S.D. total CHO oxidation rate (g·min^−1^) during 30 min submaximal cycling between* R. rosea *and placebo conditions.

**Figure 5:**
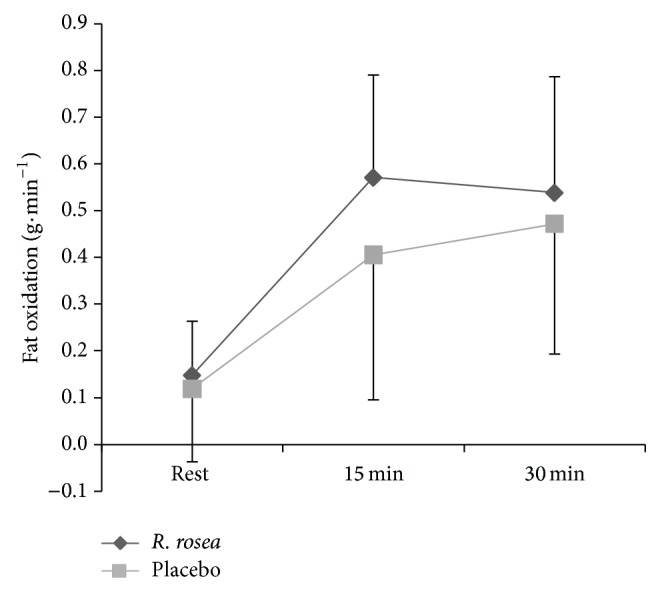
Mean ± S.D. fat oxidation rate (g·min^−1^) during 30 min submaximal cycling between* R. rosea* and placebo conditions.

Energy expenditure was significantly higher during exercise (*F* 2, 18 = 130.921, *P* < 0.001, *Pη* ^2^ = 0.936) (see Figure 3), irrespective of the trial (*F* 1, 9 = 1.198, *P* = 0.302, *Pη* ^2^ = 0.117). The rate of total carbohydrate oxidation (Figure 4) was not significantly different between trials (*F* 1, 9 = 0.317, *P* = 0.587, *Pη* ^2^ = 0.034). Carbohydrate oxidation remained relatively constant throughout exercise but was higher compared with the rest (*F* 2, 18 = 13.902, *P* < 0.001, *Pη* ^2^ = 0.770). The rate of fat oxidation (Figure 5) was higher than that at rest during exercise (*F* 1, 11 = 25.131, *P* < 0.001, *Pη* ^2^ = 0.736) but there was no significant main effect due to the substances ingested (*F* 1, 9 = 0.950, *P* = 0.355, *Pη* ^2^ = 0.095).

## 5. Discussion

Acute* R. rosea* ingestion has been reported to improve exercise performance [13, 14] in performance based tasks. Authors have suggested that this performance enhancement may result from* R. rosea's* action as an opioid producer [2, 13]. However, use of performance based exercise tasks (e.g., time trial) in prior studies makes it difficult to assess the effect of the substance ingested apart from the exercise intensity in tasks where intensity will fluctuate. Arguably, the sue of a constant load exercise protocol for 30 minutes is a more externally valid form of exercise for most individuals as it mirrors the minimum exercise recommendations for healthy adults [30]. No study to date has assessed the effects of* R. rosea* ingestion on mood, perceptions of exertion, arousal, and feelings of pleasure/displeasure during steady state exercise. In the current study, RPE and affect were measured prior to, during, and after 30-minute submaximal cycling at a moderate exercise intensity after ingestion of 3 mg·kg^−1^ *R. rosea* of placebo. This study is novel in that results demonstrated dampened RPE, greater vigor, perception of arousal, and pleasure in the presence of* R. rosea* compared to placebo. In the case of RPE and feelings of pleasure, these changes were seen at the end of the 30 min exercise bout. Thus, this data indicate that acute* R. rosea* ingestion positively modifies affect during relatively brief, steady state exercise at moderate intensity.

Previous studies have speculated that the ingestion of* R. rosea* can improve exercise performance via altered energy metabolism [16], activated by the synthesis or resynthesis of ATP in mitochondria and stimulated restorative energy processes after intense exercise [15]. However, the results of the present study suggest that altered substrate utilization is not responsible for the improved performance. The discrepancy between the findings of the present study in this respect and those of prior work [15, 16] may be due to varying preexercise fed state across studies. In the present study the Frayn [38] equation was employed to assess fat oxidation as this is the most widely applied equation for fat oxidation available. However, we acknowledge that other equations are also available [39] and may be preferable in future work. It is also important to note that there is no gold standard to validate whole body substrate oxidation but that assessment of substrate oxidation using indirect calorimetry may be prone to large errors [40]. Therefore, the acute increase in endogenous opioid production may play a role in enhancing exercise performance in the presence of* R. rosea *and is evidenced through changes in perceived exertion and exercise affect rather than changes in physiological markers.

The present study does have some limitations. The task used employed a bout of steady state exercise at a moderate intensity. Prior research has tended to employ protocols involving time trial performance [14] or time to exhaustion [13], neither of which is applicable to the typical exercising public. While the protocol employed in the present study may be valid when examining steady state exercise at a moderate intensity, it also might not fully address the typical training session undertaken by many recreational exercisers and athletes. However, given the lack of studies examining the effect of* R. rosea* on exercise performance such steady state protocols are useful in developing the extant literature in this area. We do acknowledge that by controlling exercise intensity based on a percentage of $\dot{\text{V}}{\text{O}}_{2\max}$ we are unable to determine if participants were exercising below, at, or above their lactate threshold. Exercise across these different intensities may however change affective responses [22]. This should be considered in future studies. Future research might subsequently benefit from using a standardised protocol to mimic the type of exercise session commonly undertaken in gyms (e.g., combined aerobic and resistance exercise) in order to determine if* R. rosea* ingestion is practically useful in a more ecologically valid exercise task. The current study also examined participants who were recreationally active but were not specifically cycling trained. It has been suggested that less fit individuals may experience greater fatigue and discomfort during exercise which may reduce feelings of pleasure compared to more highly trained individuals. It may therefore be useful to compare the responses of participants of different training status in order to make more conclusive statements regarding the effect of* R. rosea* on variables such as perception of exertion, arousal, and pleasure. Furthermore, a posteriori power calculations indicated a sample size of 28 would have been required to detect a difference of *P* = 0.05 at 95% power. However, due to the paucity of data on effects of* R. rosea* on exercise performance this study was exploratory in nature and subsequently used a relatively small number of participants. The current study may therefore be underpowered. Finally, the assessment of affect immediately on completion of the cycling bout might have resulted in elevated scores for feeling states due to the cessation of exercise as has been suggested previously [17]. It may be that the trajectory of pleasure and displeasure during and after exercise exhibits two distinct phases [22]. The first phase involves a decline or increase of affective responses during exercise, whereas the second phase involves an improvement or rebound of affective responses after exercise. As measures of affect were only taken on completion of the exercise bouts, the data presented here are only representative of the rebound phase of exercise in the presence of* R. rosea* and placebo. Future research should therefore attempt to assess affect during exercise in addition to immediately on cessation in order to more effectively capture the time course of affective responses to exercise following ingestion of different substances.

## 6. Conclusions

Ingestion of* R. rosea *favourably influenced RPE and exercise affect without changes in energy expenditure or substrate utilization during 30 minutes of submaximal cycling performance at moderate intensity in regularly active adults. These changes support the efficacy of acute* R. rosea* ingestion in positively enhancing psychophysiological responses to submaximal exercise performance.

## Figures and Tables

**Table 1:** Descriptive information (mean ± S.D.) of participants' height, body mass, resting heart rate, and maximal oxygen uptake.

Height (m)		Mass (kg)		Resting heart rate (bpm)		$\dot{\text{V}}{\text{O}}_{2\max}$ (mL·kg^−1^·min^−1^)	
M	S.D.	M	S.D.	M	S.D.	M	S.D.
1.72	0.03	67.7	6.3	79.8	17.3	50.5	6.6
